# Have inequalities in completeness of death registration between states in India narrowed during two decades of civil registration system strengthening?

**DOI:** 10.1186/s12939-021-01534-y

**Published:** 2021-08-30

**Authors:** Jayanta Kumar Basu, Tim Adair

**Affiliations:** 1 CRVS, UNICEF Bihar Office, Patna, India; 2 Melbourne School of Population and Global Health, The University of Melbourne, Level 5, Building 379, 207 Bouverie St, Carlton, 3053 VIC Australia

**Keywords:** Civil registration, Vital statistics, Mortality, Completeness, India, Inequalities, Socio-economic

## Abstract

**Background:**

In India the number of registered deaths increased substantially in recent years, improving the potential of the civil registration and vital statistics (CRVS) system to be the primary source of mortality data and providing more families of decedents with the benefits of possessing a death certificate. This study aims to identify whether inequalities in the completeness of death registration between states in India, including by sex, have narrowed during this period of CRVS system strengthening.

**Methods:**

Data used in this study are registered deaths by state and year from 2000 to 2018 (and by sex from 2009 to 2018) reported in the Civil Registration Reports published by the Office of Registrar General of India. Completeness of death registration is calculated using the empirical completeness method. Levels and trends inequalities in completeness are measured in each state a socio-economic indicator – the Socio-Demographic Index (SDI).

**Results:**

Estimated completeness of death registration in India increased from 58% in 2000 to 81% in 2018. Male completeness rose from 60% in 2009 to 85% in 2018 and was much higher than female completeness, which increased from 54 to 74% in the same period. Completeness remained very low in some states, particularly from the eastern (e.g. Bihar) and north-eastern regions. However, in states from the northern region (e.g. Uttar Pradesh) completeness increased significantly from a low level. There was a narrowing of inequalities in completeness according to the SDI during the period, however large inequalities between states remain.

**Conclusions:**

The increase in completeness of death registration in India is a substantial achievement and increases the potential of the death registration system as a routine source of mortality data. Although narrowing of inequalities in completeness demonstrates that the benefits of higher levels of death registration have spread to relatively poorer states of India in recent years, the continued low completeness in some states and for females are concerning. The Indian CRVS system also needs to increase the number of registered deaths with age at death reported to improve their usability for mortality statistics.

**Supplementary Information:**

The online version contains supplementary material available at 10.1186/s12939-021-01534-y.

## Background

A high quality civil registration and vital statistics (CRVS) system should be the primary source of routine mortality data to inform public health policy [1]. In India, the world’s 2^nd^ most populous country and where an estimated 17% of the world’s deaths occur, regular, timely and accurate mortality data is important not only for the national government but state and territory governments, the largest of which (Uttar Pradesh) has a population of over 200 million [2, 3]. Recent decades in India have seen substantial declines in under-five mortality, however the country faces significant health challenges, including the ongoing COVID-19 pandemic as well as continuing premature mortality from non-communicable diseases due to risk factors such as unhealthy diet and air pollution [4].

Like many countries, India does not have a complete death registration system. Instead, the Sample Registration System (SRS) has been used for many years to underpin evidence on Indian mortality. The SRS operates as a death reporting system within a sample of the population within the country. Deaths are actively notified and corrected based on a dual record system and the SRS sample is revised every ten years based on the latest census frame [5]. SRS estimates are generally valid and reliable for the country as a whole and for bigger states with more than 10 million populations, and recently the sample size of SRS has been increased to allow for estimates by natural divisions within the bigger states [6]. In recent years, however, the quality of the SRS has been questioned, with one analysis finding that it produces lower estimates of adult mortality than the death registration system in many states and also suffers from considerable uncertainty due to it being based on a sample of the population [7]. For early age mortality, data are available at the national and subnational level from household surveys such as the India National Family Health Survey (INFHS) based on retrospective detailed birth histories [2, 8, 9].

Since 2000, there has been a strong increase in not only the absolute numbers of deaths registered by the Indian CRVS system, but also the completeness when measured as a proportion of estimated total deaths. In 2000, 3.8 million deaths were registered in India, which increased to almost 7 million deaths in 2018 [10, 11]. This increase in registered deaths is substantial in the context of the 56.5 million annual global deaths [12]. With rising levels of completeness of registration, there is an increased potential for death registration data to be used as a source of routine mortality estimates, with appropriate adjustments for estimates of incompleteness [7]. Death registration also has the advantage of providing the benefits to the population with the issuance of a death certificate, such as relieving the individual from social, legal and official obligations, to enable settlement of property inheritance, and to authorize the family to collect insurance and other benefits.

The CRVS system is based upon the Registration of Births and Deaths Act, 1969, which provides for uniform law across the country on the registration of births and deaths as well as compulsory reporting and registration of all births and deaths. Initially death registration was a paper-based system, but as a strategy of further strengthening the registration of vital events the online process of birth and death registration was initiated in 2015 by the Office of the Registrar General of India. The online system of birth and death registration has provided impetus to increasing access to registration and has also introduced greater transparency and accountability to the entire process. The implementation of the births and deaths registration Act has been the responsibility of the State Governments, and this has led to inconsistencies in the functioning and reporting of the online system, which may affect inequalities in death registration. Another issue is that the data produced by the death registration system are limited because some states and territories that do not report age or sex at death and so age- and sex-specific data are not available at the national level. Some of these states are yet to use online system completely. As a result, the death registration system is not used in any international mortality estimates in India by the Global Burden of Disease (GBD) or United Nations Population Division because of non-availability of age data at the national level [2, 13].

The increase in death registration completeness in India since 2000 raises the question about whether inequalities in completeness between states have narrowed over the period. The relative level of completeness of death registration between states is important because it demonstrates the utility of the death registration system to be a source of routine subnational mortality data, particularly given the population size of some states, and also shows the extent to which the benefits of death registration are available to the population, particularly marginalized populations. In the only more populous country, China, death registration completeness in its 31 provinces in 2018 ranged from 16 to 94%, compared with the national level of 74% [14]. In India, an analysis of the percentage of children under five years in the 2015–16 INFHS reported to have had their birth registered ranged from 60% in Uttar Pradesh to 99% in several states/territories; this ranged from 64% in the poorest wealth quintile to 94% in the highest [9]. Significant inequalities in possession of a birth certificate by household wealth status have been found in most low- and middle-income countries [15]. Hence, the extent to which inequalities in death registration between states reflect socio-economic inequalities can be assessed by using a measure of socio-economic status available for each state.

This paper aims to measure whether inequalities in death registration completeness between states in India have narrowed since 2000. It does this by using the empirical completeness method to measure annual completeness for each state from 2000–18, and then assessing trends in inequalities according to the correlation of completeness with the socio-economic indicator of the Socio-Demographic Index (SDI). We also assess these trends in inequalities by sex-specific completeness, and examine the availability of age and sex data by state. These results are expected to highlight how investments in the CRVS system have been distributed across states, and where further investment is needed.

## Methods

Death registration data used in this study are registered deaths presented in the Reports on Civil Registration System in India published by the Office of Registrar General of India. We obtained data from the 2009–2018 Reports, which show numbers of deaths registered in each year from 2000–2018 by state/territory and, where available, age group and sex (sex is only reported for 2009–18) [10, 11, 16–23]. The reported deaths in the Civil Registration System reports are based on year of registration. The state of Telangana, which was officially formed out of Andhra Pradesh in 2014, is included with Andhra Pradesh in our analysis to ensure consistency of state boundaries in our analysis over times [11].

Completeness of death registration (i.e. the percentage of actual deaths that are registered) is calculated using the empirical completeness (Adair-Lopez) method [24]. This method is a statistical model that estimates completeness based on data inputs that reflect the determinants of the crude death rate; mortality level (represented by the under-five mortality rate) and population age structure (reflected by the percentage population of the aged 65 years and above). The other variables are the registered crude death rate (registered deaths divided by population multiplied by 1000) and calendar year (one version of the method also includes completeness of under-five registration, but this cannot be calculated for all states/territories due to no age data). The method has previously been used to estimate completeness of death reporting for 2,844 Chinese counties [14]. The method overcomes the limitations of other completeness estimation methods such as death distribution methods, which rely on death data by five-year age group (which are not reported for Indian death registration), are subject to inaccuracies due to implausible assumptions of population dynamics (especially at the subnational level), and the most reliable of which can only be calculated for the most recent intercensal period (which for India was 2001–11) [24]. Population data are obtained from the Population Projections for India and States 2011–36 published by the National Commission on Population (for 2011–18) and the Technical Group for Population Projection constituted by National Commission on Population who produced population projections for Indian states from 2001 onwards (for 2001–2010) [25, 26]. The under-five mortality rate data by year, sex and state/territory were obtained from a GBD analysis of national and subnational data from surveys and censuses [27].

We assessed inequalities in completeness of death registration between states according to the SDI. The SDI is a composite indicator of development that is the geometric mean of average income per person, educational attainment, and total fertility rate; it ranges from 0 to 1 [28]. The SDI has previously been used for analyses of subnational Indian epidemiological transition and under-five mortality [4, 27]. It is available for Indian states and territories from 2000–2016 and we projected for 2017 and 2018 based on the annual rate of change from 2015–16. We also considered using the Multi-dimensional Poverty Indicator (MPI) to measure inequalities; it is a measure of the proportion of a state’s population in poverty and is based on different variables of Standard of Living, Health and Education [29, 30]. However, the standard deviation of MPIs in Indian states fell sharply from 0.12 to 0.06 because there was a universal decline in poverty over the period; the smaller variation in MPI in recent years could result in a larger negative coefficient when predicting completeness and hence incorrectly show widening inequalities. The standard deviation of the SDI, in contrast, only changed from 0.064 to 0.066 between 2000 and 2018. Other indicators such as literacy and malnutrition among children were also investigated as indicators of inequalities but did not show as strong an association as SDI.

Inequalities were measured using a linear regression of completeness for each state with a covariate of SDI. For each year, the regression coefficient of SDI was used as our indicator of the extent of inequalities in completeness between states. SDI is expected to have a positive relationship with completeness, so a narrowing of inequalities would be demonstrated by the coefficient falling over time; in other words, if SDI is less important in predicting completeness, then inequalities in completeness according to states’ socio-economic development have fallen. Given the large variation in the size of the populations of Indian states, we also conducted the regressions for SDI and completeness for both sexes weighted by population, to assess if it had any impact on trends.

## Results

**Fig. 1:**
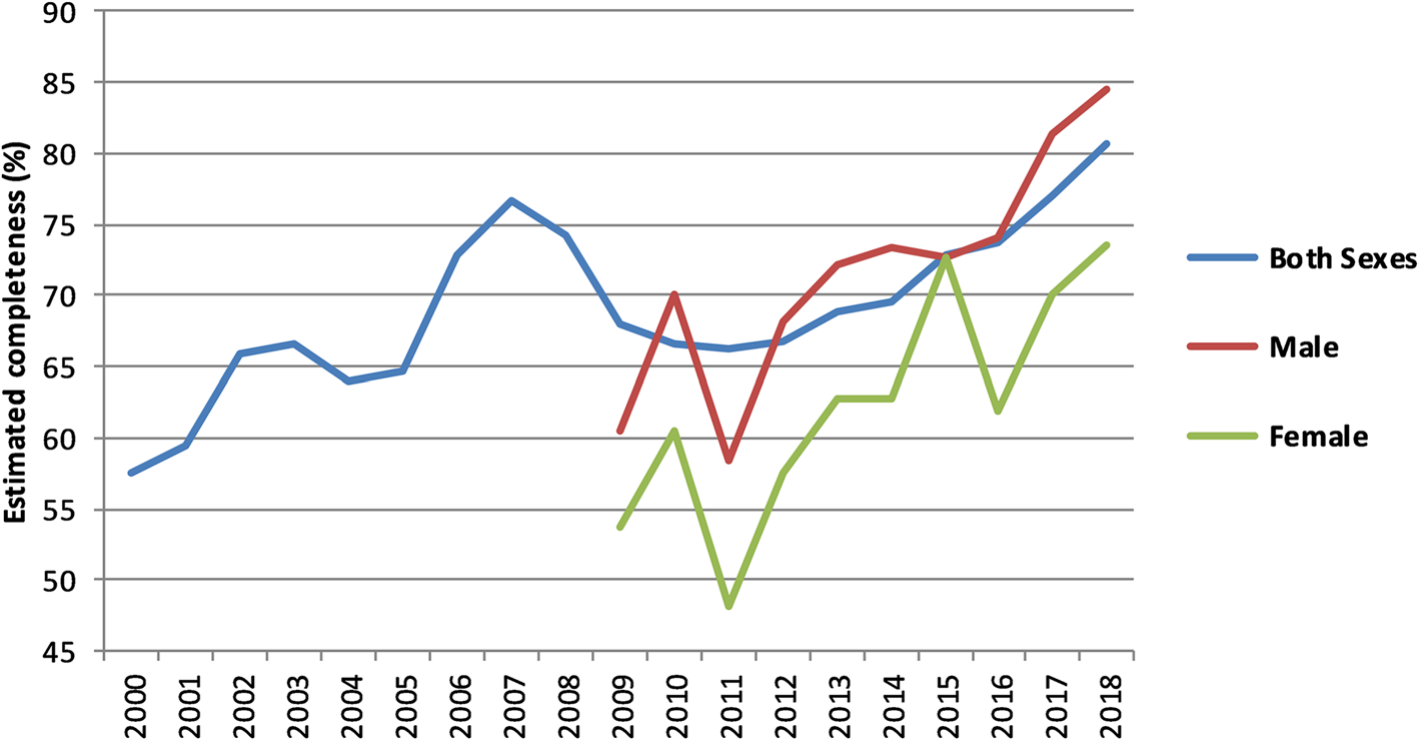
Estimated completeness of death registration (%), both sexes, male and female, India, 2000–2018

Estimated completeness of death registration in India for both sexes increased from 58% in 2000 to 81% in 2018. There was a sharp increase from 2000 to 2007, followed by a decline and then another increase in recent years (Fig. 1). Male completeness was much higher than female completeness in most years; male completeness rose from 60% in 2009 to 85% in 2018, while female completeness increased from 54 to 74% in the same period.

**Fig. 2:**
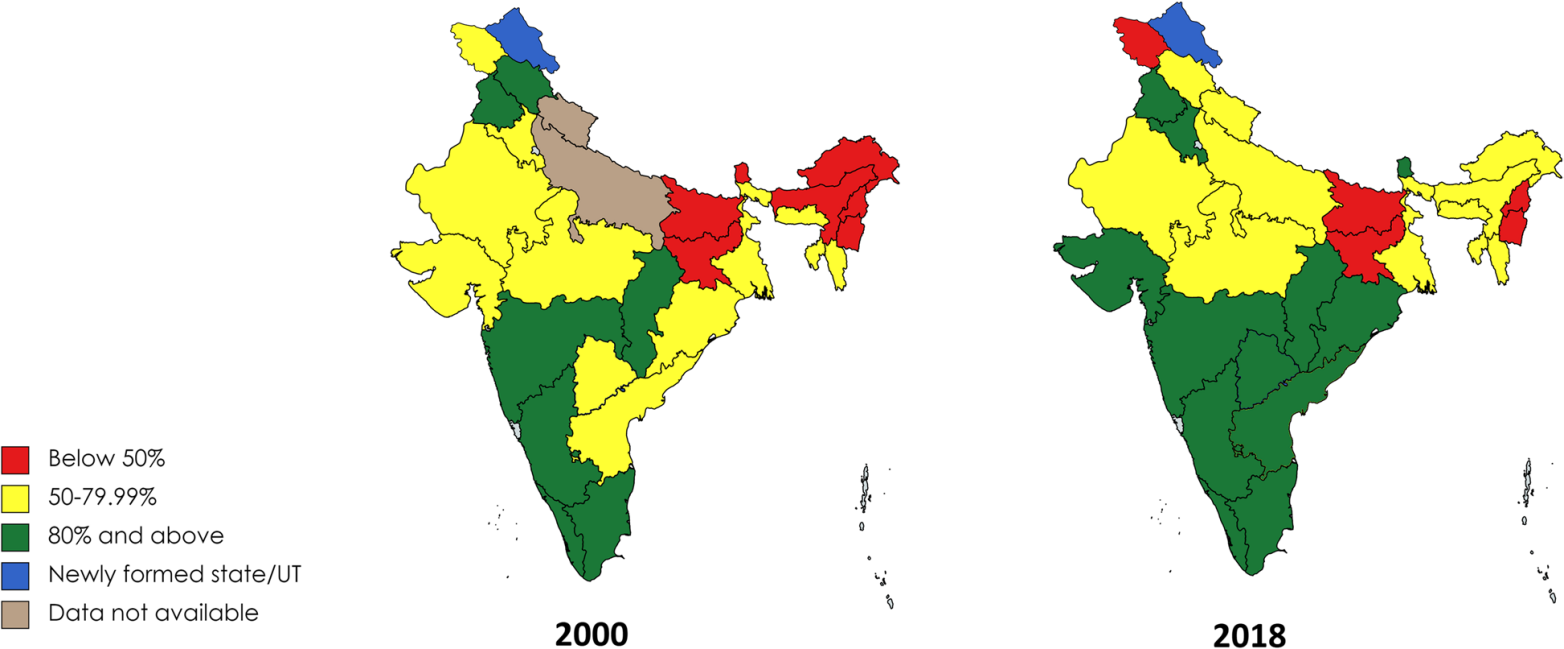
Estimated Completeness of death registration in Indian States, both sexes, 2000 and 2018

Table 1 shows completeness of registration for both sexes in each state as well as availability of registered death data by age group and sex for selected years. Though estimates indicate an increase in death registration completeness at the national level over the years during 2000 to 2018, the trend and level of the rise at the sub-national level was unevenly distributed (Table 1, Figs. 1 and 2, Table A1). The level of increase in completeness was very low in some of the states, particularly from eastern (Bihar, Jharkhand) and north-eastern (Manipur, Nagaland) regions of the country. States from the southern region (Kerala, Karnataka, Tamil Nadu) had consistently higher completeness throughout the period. In the western part of the country, Goa and Maharashtra maintained a higher than average level of completeness, whereas for Gujarat it increased from 67.4 percent in 2000 to 87.7 percent in 2018. In some states from the northern region (Uttar Pradesh, Uttarakhand) the level of completeness increased significantly from a low level in the earlier years, whereas in Jammu & Kashmir it declined from 2000 to 2018. Differences in male and female completeness are particularly large in some states (Figure A1, Tables A2-A3). For example, in the northern states of Rajasthan completeness in 2018 was 87% for males and 62% for females and in Arunachal Pradesh for males it was 70 and 49% for females (Tables A2-A3).

**Table 1:** India: Completeness of death registration (both sexes) availability of age and sex data, 2000, 2005, 2010, 2015, 2018

Region	States	Population (2018) - Both Sexes (‘000 s)	2000	2005	2010	2015	2018
**Northern**	NCT of Delhi	22,523	88.9	91.7	96.6**	95.8**	98.6**
	Haryana	28,253	75.8	70.2	81.8*	85.7*	88.9*
	Himachal Pradesh	7,206	80.2	84.1	85.6**	82.2*	79.4**
	Jammu & Kashmir	12,665	54.4	50.3	50.6**	49.7**	49.3**
	Punjab	29,625	84.8	84.3	89.2**	90.1**	90.6**
	Rajasthan	74,884	52.6	63.9	74.0**	77.5**	79.8**
	Uttar Pradesh	224,829	DNA	16.1	47.3*	35.4*	53.6*
	Uttarakhand	10,887	DNA	49.5	48.4*	69.3*	72.7*
**Southern**	Andhra Pradesh	89,691	64.6	61.1	69.0**	80.6**	85.2**
	Karnataka	63,435	89.1	88.0	86.5**	86.4**	88.9**
	Kerala	36,062	93.9	95.9	97.8**	97.5**	97.3**
	Tamil Nadu	70,047	84.1	89.6	92.8**	96.0**	94.0**
**Western**	Goa	2,068	95.0	96.2	97.4**	97.2**	98.8**
	Gujarat	67,222	67.4	69.4	80.1**	88.0	87.7**
	Maharashtra	122,926	81.1	80.3	86.6*	85.8**	84.3*
**Eastern**	Bihar	106,192	12.2	22.4	15.3**	26.2*	26.0*
	Jharkhand	34,483	27.8	48.8	48.9*	59.9*	42.6*
	Odisha	43,132	77.6	79.0	81.2**	86.0**	83.0**
	West Bengal	95,109	60.4	53.5	59.6*	72.6*	79.4**
**Central**	Chhattishgarh	26,488	81.6	80.2	60.5*	81.8**	81.2**
	Madhya Pradesh	80,042	61.2	58.3	56.0**	53.0**	72.2**
**North Eastern**	Arunachal Pradesh	1,341	23.3	24.0	46.4*	62.8**	50.4**
	Assam	33,166	24.7	40.8	43.4*	47.3**	56.8**
	Manipur	2,646	32.5	43.6	40.3**	33.4*	36.2**
	Meghalaya	2,832	78.2	60.1	80.2**	82.5**	72.9**
	Mizoram	1,085	77.6	83.5	81.7**	86.2**	72.9**
	Nagaland	2430	47.1	55.8	68.7**	25.0**	19.4**
	Sikkim	660	42.8	79.4	87.0**	90.7*	91.2*
	Tripura	3,906	65.9	DNA	35.7*	44.5**	61.3**

Source: Civil Registration System (CRS) Report, ORGI, Govt. of India; Population Projection by ORGI, Govt. of India

*DNA* Data Not Available * Either age or sex data available. ** Both age and sex data available. If no asterisk(s), neither age nor sex data available

As shown in Table 1, the death registration data are limited – no sex data were available before 2009 and no age at death data for some states/union territories were available throughout the entire period; hence, no age/sex at death data at national level is available. Seven states with a combined population of 528 million (or 41% of India’s population) didn’t report deaths by age. However, some improvement in reporting of age-sex data of the deceased by the states was observed over the years. The states in the southern region of the country have made significant improvement in reporting of age-sex death registration data. Though some improvement in availability of age-sex data was observed in the states from central, northern and north-eastern region of India, the states in the eastern part of the country are still lagging others.

**Fig. 3:**
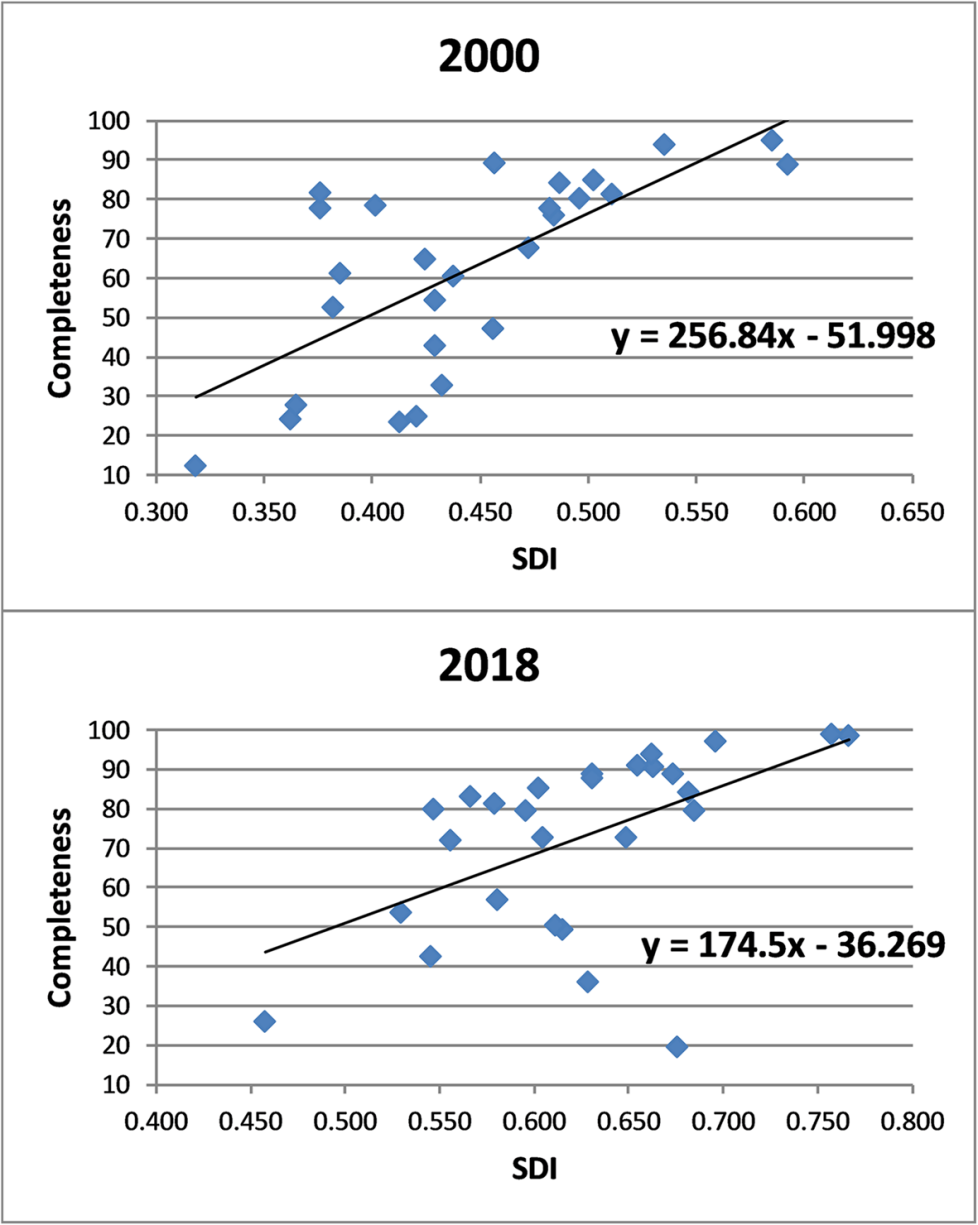
SDI by completeness of death registration, both sexes, India, 2000 and 2018

**Fig. 4:**
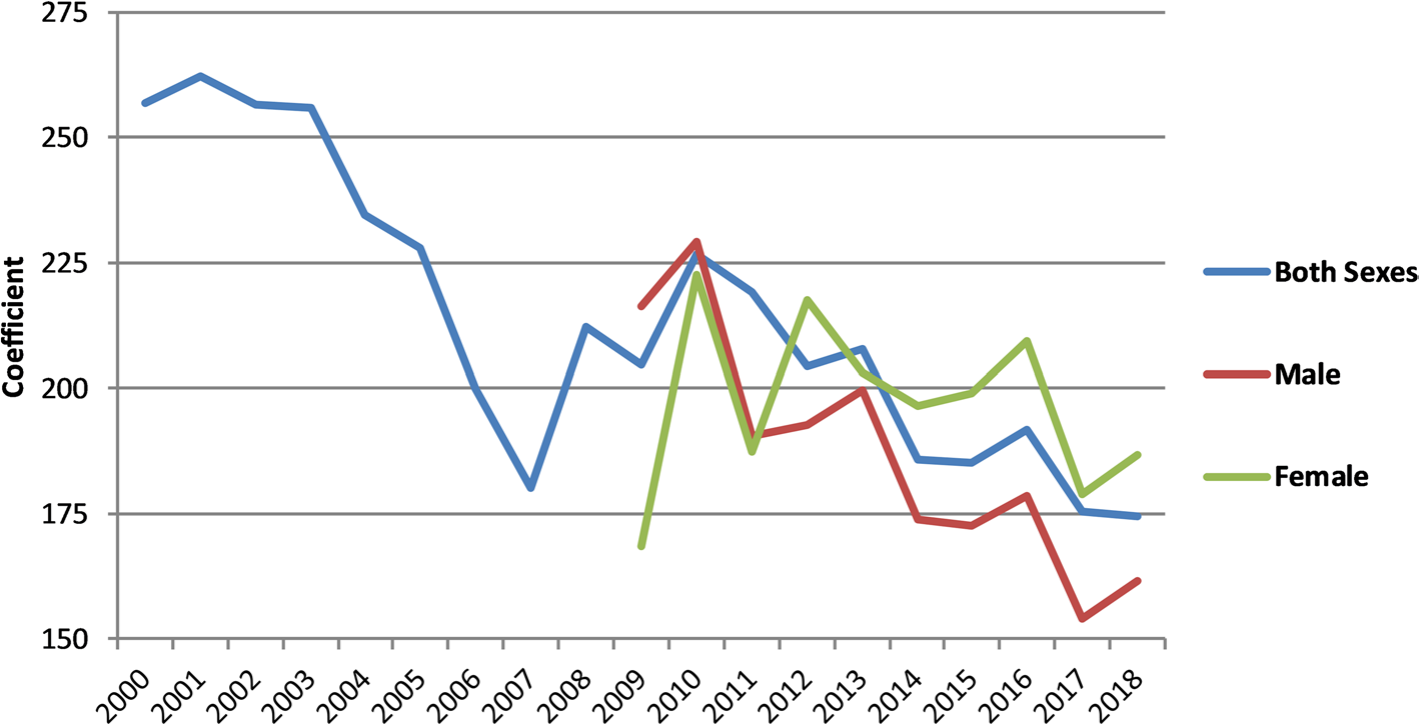
Trends in coefficient of SDI (from regression to predict death registration completeness), both sexes, male and female, 2000–2018, India

Across Indian states, there has been an increase in the SDI, and a slight decline in the standard deviation of the SDI across states relative to average SDI (Table A4). Figure 3 shows that there is a strong positive correlation between death registration completeness for both sexes and SDI across the Indian states. That is, states with lower SDI overall had a lower completeness than the states with a higher SDI. However, the strength of this association weakened over the period. As the completeness in many states increased above 50%, compared with states with a higher level in 2000, inequalities narrowed. The SDI coefficient (SDI vis-à-vis predicted completeness) for both sexes declined from 257 in 2000 to 175 in 2018 (Fig. 4). There was a sharp decline in the coefficient to 180 in 2007 before increasing and then declining again. Again, when the coefficients were determined from a population-weighted regression they declined, from 333 in 2000 to 245 in 2018; there was a sharp decline to 2007, followed by a rise and then another fall. Interestingly, the trend in male and female inequalities differed from 2009 to 2018. Inequalities for males declined from 216 to 162, but for females increased from 169 to 187 (although with some year-to-year variation) (Figures A2-A3).

## Discussion

The additional 3.2 million annual registered deaths between 2000 and 2018, an increase in estimated completeness from 58 to 81%, is a substantial achievement and dramatically increases the potential of the death registration system to be used as a routine source of mortality data. These changes have occurred with a narrowing of inequalities in completeness between states measure by the SDI, whether it was measured weighted by population size or not. Both male and female completeness increased from 2009–18, but with SDI for females remaining steady, for males narrowing. The largest increases in completeness were found in Rajasthan, Gujarat, Assam, Arunachal Pradesh and Sikkim, and the largest state, Uttar Pradesh, where completeness increased from 18% in 2002 to 54% in 2018. The trend in completeness is characterized by an initial increase to 2007, followed by a dip and then increase in recent years.

The narrowing of inequalities in completeness demonstrates that the benefits of higher levels of death registration, including for mortality statistics and individuals and families, have spread to relatively poorer states of India in recent years. However, the continued low completeness in some states, such as the poorest state Bihar, as well as in Nagaland where there was a substantial decline in completeness over the period, are concerning. Low female compared with male registration in many states is also an area where much improvement in the system is needed, including in Rajasthan and Arunachal Pradesh. The Indian death registration system also needs to increase the number of deaths with age reported to improve their usability for mortality statistics. In 2018, seven states with comprising 41% of India’s population didn’t report deaths by age. Even where deaths are reported they are in 10-year age groups, whereas calculation of life expectancy and many mortality indicators require deaths reported in five-year age groups. Although not a focus of our study, child death registration is very low in India, with another study estimating that less than one-third of deaths under five years are registered [7].

During the COVID-19 pandemic, the lack of complete and timely death registration data in India have prevented timely measurement of excess mortality and potentially masks the true extent of its impact in some states more than others. The extent of variation in the death registration in India across the states, and importantly the varied progress made by the individual states over the decade, reinforces that the steps to improve death registration will have to be addressed at the state level, as generic recommendations to improve completeness may not be applicable across all states. Lessons could be learnt from the states which have made progress in completeness for the states which are lagging behind. In general, some facilitators for death registration, such as access to insurance funds and succession of property owned by the deceased, and barriers to death registration, such as discontinuation of the government pension post death of a retired public sector employee or poor inclination to register death of a child, are acknowledged in India [31]. Other issues with death registration in poorer states include them having a poor system of initial notification of deaths, not having an institutionalized mechanism for death registration (especially at the village level), having insufficient monitoring mechanisms, lacking a computerized death registration system, there being limited training and sensitization workshops at the community level, lacking human resources in vital statistics at the state and district level, and registration units in rural areas often not functioning properly [32]. To increase completeness, these states can improve awareness in the community of the benefits of death registration, strengthen the system of initial notification of deaths, implement better monitoring of death registration data, strengthen training of registration staff, and make registration offices more readily accessible [32]. Death registration was also shown to be higher for males than females; this relative under-registration of female deaths is likely because India is a patriarchal society with the succession rights linked to males [33].

There are some limitations with our study. Deaths are published according to year of registration, so are subject to annual fluctuations caused by both delayed registrations and also collation of registered deaths. Hence, annual levels of completeness may be affected by reporting efficiencies completeness by year of registration may differ from that reported by year of occurrence. However, the longer-term trend in increased completeness is clear, irrespective of whether year of registration of occurrence is used. Also, even though our study found that many states do not report detailed age data for registered deaths, does not mean they are not available in the data for policymakers. However, good practice is for these to be published, to enable external analysts to use the data. The measures of completeness used in the study are estimates; however the estimate of completeness in 2018 of 81% was only slightly higher than that calculated according the GBD estimated deaths of 75% [2]. Finally, the summary socio-economic measures only show state-level and not individual socio-economic differences in death registration; it is likely that differences by individual socio-economic and also by smaller area of enumeration, as shown in Chinese counties, would be larger than according to state [14].

Concentrated effort is needed in the assessment of strengths and weaknesses at the subnational level of not only CRVS processes in India but the data it produces is essential to ensure that improvements in completeness are continued. Given that India accounts for one-in-six of the world’s deaths, increased routine reporting of these deaths will help with understanding of global health trends. Furthermore, improved cause of death data through both Medical Certification of Causes of Death for facility deaths and routine verbal autopsy for community deaths will help improve understanding of national and subnational cause of death patterns and enable more reliable computation of health indicators such as the Sustainable Development Goals by local analysts [34, 35].

## Conclusions

The narrowing of inequalities in completeness according to the SDI demonstrates that the benefits of higher levels of death registration have spread to relatively poorer states of India in recent years. However, the continued low completeness in some states, such as the poorest state Bihar, as well as in Nagaland where there was a substantial decline in completeness over the period, are concerning. Low female compared with male registration in many states is also an area where much improvement in the system is needed, including in Rajasthan and Arunachal Pradesh. The Indian death registration system also needs to increase the number of deaths with age reported to improve their usability for mortality statistics.

## Supplementary Information

**Additional file 1.** Additional tables and figures

## Data Availability

The data that support the findings are publicly available in the references listed in the article.

## Abbreviations

CRVS: Civil registration and vital statistics
GBD: Global Burden of Disease
INFHS: India National Family Health Survey
MPI: Multi-dimensional Poverty Indicator
SDI: Socio-Demographic Index
SRS: Sample Registration System
