# Supplementary material for: Have inequalities in completeness of death registration between states in India narrowed during two decades of civil registration system strengthening?
Source: Int J Equity Health. 2021 Aug 30;20:195. doi: 10.1186/s12939-021-01534-y (PMC8403822; doi:10.1186/s12939-021-01534-y)
Supplement: Supplementary file 1 — Additional file 1. Additional tables and figures [file 12939_2021_1534_MOESM1_ESM.docx]

**Additional File 1: Additional tables and figures**

**Figure A1: State-wise Predicted Completeness of Death Registration (empirical method) (%), Male and Female, India, 2009 and 2018**

**Male**


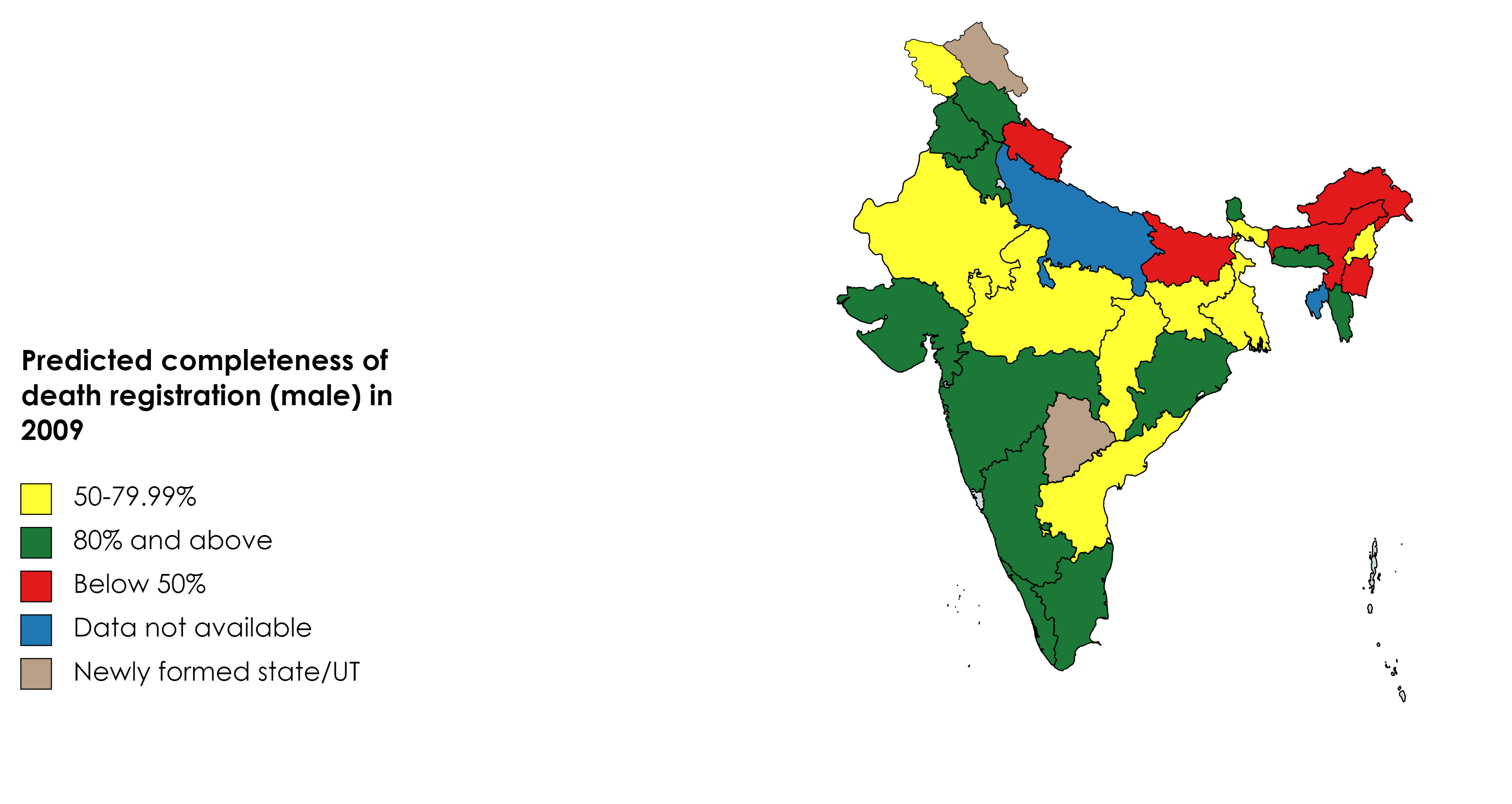


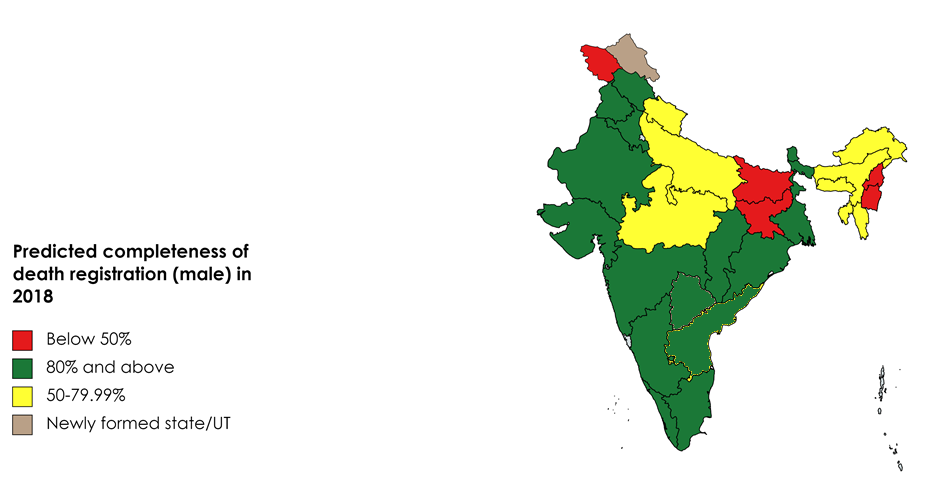


**Figure A1 (contd.):**


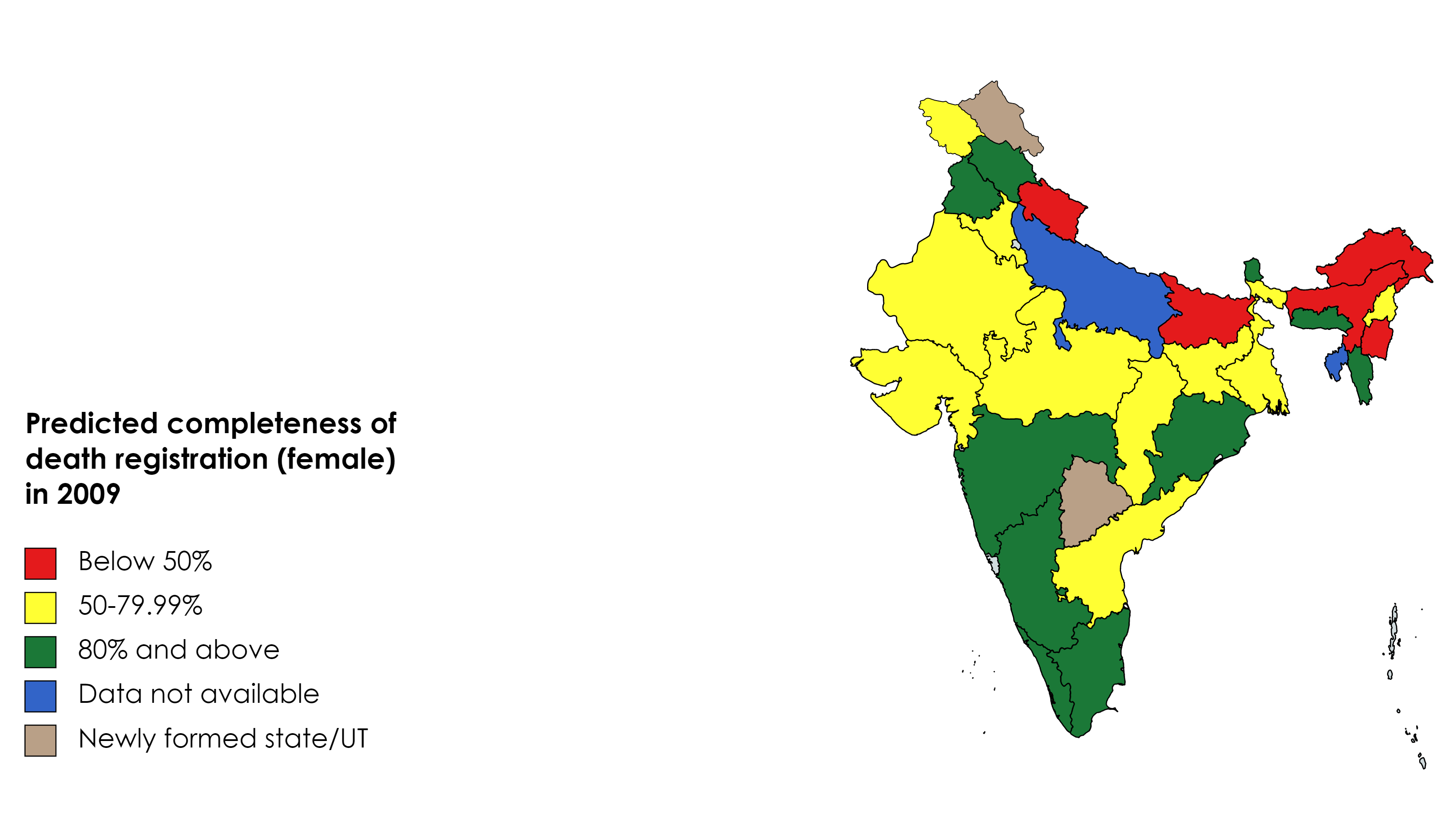


**Female**


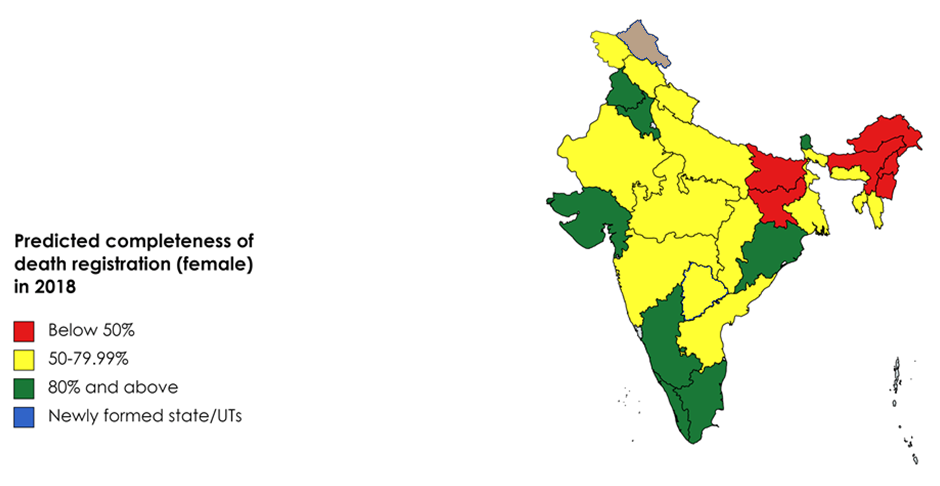


**Figure A2: SDI vis-à-vis Predicted Completeness for Males, India, 2009 and 2018**

**Figure A3: SDI vis-à-vis Predicted Completeness for Females, India, 2009 and 2018**

**Figure A4: Trends in population-weighted coefficient of SDI (from regression to predict state-level death registration completeness), 2000-2018, India**

**Table A1: Predicted Completeness (%) in India and the States, 2000 – 2018 (Both Sexes)**

| **Country/Sub-national (States)** | **2000** | **2001** | **2002** | **2003** | **2004** | **2005** | **2006** | **2007** | **2008** | **2009** | **2010** | **2011** | **2012** | **2013** | **2014** | **2015** | **2016** | **2017** | **2018** |
| --- | --- | --- | --- | --- | --- | --- | --- | --- | --- | --- | --- | --- | --- | --- | --- | --- | --- | --- | --- |
| **India** | **57.6** | **59.4** | **65.8** | **66.6** | **64.0** | **64.6** | **72.8** | **76.7** | **74.3** | **67.9** | **66.5** | **66.0** | **66.8** | **68.8** | **69.6** | **72.9** | **73.7** | **77.0** | **80.6** |
| **1. National Capital Territory (NCT) of Delhi** | 88.9 | 89.2 | 90.5 | 90.6 | 89.2 | 91.7 | 92.7 | 92.9 | 93.8 | 94.2 | 96.6 | 95.2 | 93.3 | 91.2 | 95.6 | 95.8 | 97.3 | 96.9 | 98.6 |
| **2. Haryana** | 75.8 | 75.9 | 76.1 | 79.7 | 76.9 | 70.2 | 74.8 | 79.3 | 81.8 | 79.1 | 81.8 | 83.0 | 83.9 | 83.7 | 84.6 | 85.7 | 88.5 | 87.2 | 88.9 |
| **3. Himachal Pradesh** | 80.2 | 83.7 | 84.0 | 85.3 | 83.2 | 84.1 | 86.5 | 85.7 | 87.5 | 86.7 | 85.6 | 86.3 | 86.5 | 80.6 | 80.5 | 82.2 | 74.4 | 79.1 | 79.4 |
| **4. Jammu & Kashmir** | 54.4 | 45.4 | 50.0 | 50.7 | 50.4 | 50.3 | 50.8 | 50.0 | 49.2 | 54.7 | 50.6 | 51.0 | DNA | 51.5 | 52.1 | 49.7 | 49.4 | 51.2 | 49.3 |
| **5. Punjab** | 84.8 | 86.1 | 85.9 | 87.6 | 82.8 | 84.3 | 85.0 | 87.1 | 88.7 | 88.8 | 89.2 | 90.0 | 90.1 | 89.8 | 90.7 | 90.1 | 92.2 | 91.6 | 90.6 |
| **6. Rajasthan** | 52.6 | 61.6 | 67.0 | 62.5 | 68.4 | 63.9 | 69.7 | 70.0 | 70.0 | 70.9 | 74.0 | 72.2 | 72.3 | 76.8 | 76.5 | 77.5 | 78.4 | 79.2 | 79.8 |
| **7. Uttar Pradesh** | DNA | DNA | 17.8 | 15.7 | 14.4 | 16.1 | 45.2 | 73.7 | 59.4 | 53.7 | 47.3 | 42.1 | 34.3 | 42.3 | 38.6 | 35.4 | 30.1 | 28.3 | 53.6 |
| **8. Uttarakhand** | DNA | DNA | DNA | DNA | 43.1 | 49.5 | 41.6 | 48.7 | 46.5 | 49.6 | 48.4 | 49.0 | 54.2 | 53.2 | 55.2 | 69.3 | 74.2 | 56.2 | 72.7 |
| **9. Andhra Pradesh** | 64.6 | 65.6 | 66.5 | 66.5 | 65.7 | 61.1 | 69.4 | 72.9 | 70.1 | 72.9 | 69.0 | 70.0 | 68.5 | 69.2 | 77.4 | 79.1 | 87.1 | 86.3 | 88.1 |
| **10. Karnataka** | 89.1 | 89.9 | 88.5 | 88.4 | 86.1 | 88.0 | 89.8 | 89.2 | 87.9 | 87.7 | 86.5 | 87.0 | 88.4 | 88.5 | 88.4 | 86.4 | 88.9 | 88.9 | 88.9 |
| **11. Kerala** | 93.9 | 94.4 | 94.5 | 95.3 | 95.5 | 95.9 | 96.8 | 97.5 | 96.7 | 97.3 | 97.8 | 98.0 | 97.5 | 98.0 | 97.6 | 97.5 | 97.6 | 97.8 | 97.3 |
| **12. Tamil Nadu** | 84.1 | 87.4 | 88.7 | 89.9 | 88.8 | 89.6 | 91.5 | 90.8 | 90.6 | 92.1 | 92.8 | 93.0 | 93.8 | 94.0 | 95.5 | 96.0 | 95.9 | 96.3 | 94.0 |
| **13. Goa** | 95.0 | 95.8 | 96.6 | 96.3 | 96.3 | 96.2 | 96.5 | 95.7 | 94.7 | 95.0 | 97.4 | 97.8 | 97.1 | 97.1 | 97.5 | 97.2 | 97.5 | 97.4 | 98.8 |
| **14. Gujarat** | 67.4 | 72.0 | 67.5 | 72.6 | 75.3 | 69.4 | 78.7 | 79.7 | 80.3 | 80.8 | 80.1 | 80.2 | 76.8 | 80.5 | 81.1 | 88.0 | 88.6 | 85.6 | 87.7 |
| **15. Maharashtra** | 81.1 | 81.9 | 84.1 | 86.2 | 76.5 | 80.3 | 82.1 | 84.7 | 86.0 | 85.5 | 86.6 | 84.3 | 85.2 | 85.8 | 85.9 | 85.8 | 86.6 | 84.3 | 84.3 |
| **16. Bihar** | 12.2 | 12.8 | 13.4 | 14.6 | 18.9 | 22.4 | 24.6 | 24.9 | 23.6 | 19.4 | 15.3 | 20.0 | 33.0 | 19.6 | 20.4 | 26.2 | 23.3 | 35.0 | 26.0 |
| **17. Jharkhand** | 27.8 | 28.3 | 32.1 | 36.9 | 42.9 | 48.8 | 50.7 | 51.2 | 48.4 | 53.6 | 48.9 | 52.0 | 49.6 | 50.2 | 58.8 | 59.9 | 58.1 | 52.4 | 42.6 |
| **18. Odisha** | 77.6 | 77.4 | 79.0 | 78.8 | 75.1 | 79.0 | 77.7 | 79.8 | 81.7 | 83.8 | 81.2 | 80.2 | 82.2 | 80.9 | 85.3 | 86.0 | 88.8 | 86.1 | 83.0 |
| **19. West Bengal** | 60.4 | 63.6 | 64.3 | 63.3 | 59.6 | 53.5 | 54.0 | 56.0 | 67.9 | 66.0 | 59.6 | 68.2 | 74.0 | 79.9 | 70.8 | 72.6 | 77.9 | 77.9 | 79.4 |
| **20. Chhattisgarh** | 81.6 | 76.6 | 80.9 | 79.6 | 76.2 | 80.2 | 79.7 | 81.9 | 59.5 | 61.2 | 60.5 | 60.0 | 72.7 | 75.7 | 83.5 | 81.8 | 85.9 | 84.3 | 81.2 |
| **21. Madhya Pradesh** | 61.2 | 59.8 | 60.0 | 64.1 | 59.1 | 58.3 | 63.8 | 60.2 | 53.4 | 57.2 | 56.0 | 61.0 | 61.6 | 63.9 | 61.8 | 53.0 | 58.5 | 65.1 | 72.2 |
| **22. Arunachal Pradesh** | 23.3 | 27.8 | 23.0 | 21.8 | 25.4 | 24.0 | 25.4 | 29.0 | 29.1 | 30.1 | 46.4 | 34.3 | 36.9 | 35.4 | 47.6 | 62.8 | 48.5 | 44.9 | 50.4 |
| **23. Assam** | 24.7 | 28.3 | 33.7 | 33.8 | 33.3 | 40.8 | 42.8 | 32.6 | 32.7 | 42.6 | 43.4 | 46.0 | 45.2 | 42.0 | 46.7 | 47.3 | 54.1 | 59.3 | 56.8 |
| **24. Manipur** | 32.5 | 29.5 | 41.8 | 34.6 | 39.1 | 43.6 | 39.1 | 41.2 | 30.2 | 40.3 | 40.3 | 40.7 | 40.7 | 49.5 | 54.5 | 33.4 | DNA | 38.2 | 36.2 |
| **25. Meghalaya** | 78.2 | 62.0 | 55.7 | 55.4 | 77.2 | 60.1 | 82.2 | 75.4 | 72.2 | 87.1 | 80.2 | 82.2 | 79.7 | 76.1 | 79.9 | 82.5 | DNA | 77.0 | 72.9 |
| **26. Mizoram** | 77.6 | 82.4 | 84.6 | 85.9 | 84.5 | 83.5 | 82.3 | 82.3 | 86.3 | 88.3 | 81.7 | 82.0 | 87.6 | 84.7 | 86.6 | 86.2 | 85.6 | 79.0 | 72.9 |
| **27. Nagaland** | 47.1 | 51.3 | 56.1 | 48.9 | 53.7 | 55.8 | 62.5 | 53.9 | 62.4 | 56.2 | 68.7 | 69.3 | 67.9 | 67.0 | 25.3 | 25.0 | 25.6 | 24.7 | 19.4 |
| **28. Sikkim** | 42.8 | 53.1 | 67.1 | 79.1 | 80.7 | 79.4 | 80.0 | 79.0 | 81.0 | 85.0 | 87.0 | 87.0 | 90.8 | 90.4 | 90.5 | 90.7 | 91.3 | 90.4 | 91.2 |
| **29. Tripura** | 65.9 | 65.9 | 37.0 | 49.5 | 66.5 | DNA | 63.0 | DNA | DNA | DNA | 35.7 | 40.1 | 42.1 | 53.5 | 41.3 | 44.5 | 45.8 | 61.3 | 61.3 |

DNA-Death Registration data Not Available

1-8: States from northern region of India

9-12: States from southern region of India

13-15: States from western region of India

16-19: States from eastern region of India

20-21: States from central region of India

22-29: States from north-eastern region of India

**Table A2: Predicted Completeness (%) in India and the States, 2009 – 2018 (Male)**

| **Country/Sub-national (States)** | **2009** | **2010** | **2011** | **2012** | **2013** | **2014** | **2015** | **2016** | **2017** | **2018** |
| --- | --- | --- | --- | --- | --- | --- | --- | --- | --- | --- |
| **India** | **60.4** | **70.0** | **58.4** | **68.2** | **72.1** | **73.4** | **72.7** | **74.0** | **81.3** | **84.5** |
| **National Capital Territory (NCT) of Delhi** | 93.9 | 96.6 | 95.0 | 93.3 | 91.7 | 95.7 | 95.7 | 97.2 | 96.7 | 98.5 |
| **Haryana** | 84.3 | 85.6 | 86.9 | 87.4 | 87.6 | 88.4 | 89.3 | 91.4 | 90.2 | 91.6 |
| **Himachal Pradesh** | 88.1 | 87.7 | 87.7 | 88.2 | 83.0 | 83.2 | 84.6 | 78.4 | 82.0 | 81.5 |
| **Jammu & Kashmir** | 51.5 | 49.6 | 49.5 | DNA | 50.9 | 54.5 | 54.0 | 53.5 | 53.9 | 49.5 |
| **Punjab** | 90.2 | 91.1 | 91.1 | 91.3 | 90.5 | 91.2 | 91.1 | 93.1 | 92.0 | 91.4 |
| **Rajasthan** | 75.2 | 80.5 | 74.8 | 76.0 | 82.1 | 82.8 | 84.0 | 84.5 | 86.3 | 87.0 |
| **Uttar Pradesh** | DNA | DNA | DNA | 30.7 | 43.7 | 40.6 | 37.6 | 32.6 | 30.7 | 53.0 |
| **Uttarakhand** | 54.1 | 55.1 | 53.9 | 59.2 | 57.5 | 60.5 | 74.4 | 78.0 | 63.5 | 77.0 |
| **Andhra Pradesh** | 73.9 | 70.8 | 71.9 | 70.4 | 69.2 | 77.4 | 79.1 | 87.1 | 86.3 | 88.1 |
| **Karnataka** | 89.8 | 89.3 | 89.0 | 90.5 | 91.1 | 90.7 | 88.8 | 91.4 | 93.9 | 92.5 |
| **Kerala** | 97.8 | 98.0 | 98.3 | 97.6 | 98.5 | 98.1 | 97.9 | 98.0 | 96.5 | 97.9 |
| **Tamil Nadu** | 93.5 | 93.8 | 93.1 | 94.1 | 95.0 | 96.1 | 96.4 | 96.4 | 96.6 | 94.9 |
| **Goa** | 94.8 | 97.2 | 96.7 | 97.3 | 97.4 | 97.6 | 97.3 | 97.5 | 97.4 | 98.8 |
| **Gujarat** | 84.3 | 84.0 | 83.6 | 82.0 | 84.2 | 84.8 | DNA | DNA | 87.5 | 89.4 |
| **Maharashtra** | 86.5 | 87.9 | 86.1 | 87.1 | 87.6 | 87.7 | 87.6 | 87.9 | 85.9 | 86.2 |
| **Bihar** | 8.7 | 11.9 | DNA | DNA | 23.7 | 24.8 | 32.8 | 28.1 | 40.3 | 29.5 |
| **Jharkhand** | 56.6 | 51.3 | DNA | 55.7 | 54.3 | 63.9 | 59.5 | 62.7 | 56.3 | 46.6 |
| **Odisha** | 81.9 | 80.3 | 79.2 | 80.8 | 80.4 | 84.3 | 85.6 | 87.7 | 84.1 | 80.6 |
| **West Bengal** | 68.6 | 62.8 | 70.8 | 76.3 | 78.2 | 73.2 | 74.2 | 79.7 | 79.0 | 80.7 |
| **Chhattisgarh** | 67.1 | 66.6 | 65.7 | 77.8 | 80.2 | 84.3 | 82.1 | 84.8 | 84.6 | 82.6 |
| **Madhya Pradesh** | 58.0 | 57.2 | 62.4 | 62.5 | 64.5 | 62.5 | 55.8 | 63.1 | 70.4 | 77.9 |
| **Arunachal Pradesh** | 38.5 | 55.0 | 37.2 | 42.5 | 38.6 | 57.5 | 74.4 | 55.4 | 62.8 | 70.0 |
| **Assam** | 40.5 | 42.0 | 41.7 | 42.8 | 40.9 | 58.9 | 59.3 | 62.5 | 56.3 | 62.7 |
| **Manipur** | 41.7 | 43.0 | 42.9 | 42.6 | 58.7 | 65.0 | 34.5 | DNA | 36.2 | 40.6 |
| **Meghalaya** | 83.7 | 79.0 | 79.6 | 81.2 | 73.7 | 79.8 | 81.6 | DNA | 76.7 | 72.2 |
| **Mizoram** | 88.9 | 83.7 | 85.0 | 90.1 | 88.0 | 89.0 | 89.1 | 88.3 | 82.9 | 77.9 |
| **Nagaland** | 53.8 | 64.2 | 64.2 | 62.9 | 61.6 | 28.3 | 27.3 | 27.4 | 26.0 | 19.2 |
| **Sikkim** | 85.0 | 86.7 | 88.0 | 89.7 | 89.6 | 89.4 | 90.0 | 90.9 | 88.0 | 87.3 |
| **Tripura** | DNA | 34.1 | 38.5 | 47.6 | 47.8 | 46.6 | 48.2 | 52.1 | 71.2 | 71.4 |

DNA: Data Not Available

**Table A3: Predicted Completeness (%) in India and the States, 2009 – 2018 (Female)**

| **Country/Sub-national (States)** | **2009** | **2010** | **2011** | **2012** | **2013** | **2014** | **2015** | **2016** | **2017** | **2018** |
| --- | --- | --- | --- | --- | --- | --- | --- | --- | --- | --- |
| **India** | **53.7** | **60.4** | **48.1** | **57.6** | **62.8** | **62.8** | **72.7** | **61.8** | **70.1** | **73.6** |
| **National Capital Territory (NCT) of Delhi** | 93.1 | 95.1 | 93 | 91.1 | 88.5 | 94.1 | 94.5 | 96.3 | 95.9 | 97.8 |
| **Haryana** | 66.5 | 68.1 | 71.1 | 73.3 | 72.9 | 73.6 | 74.9 | 79.0 | 78.3 | 80.3 |
| **Himachal Pradesh** | 82.2 | 80.5 | 80.7 | 81.3 | 74.6 | 74.5 | 76.4 | 67.1 | 72.9 | 74.5 |
| **Jammu & Kashmir** | 59.6 | 54.5 | 54.2 | DNA | 53.8 | 50.4 | 45.6 | 45.7 | 49.2 | 51 |
| **Punjab** | 83.0 | 83.1 | 83.9 | 85.3 | 86.5 | 87.6 | 86.3 | 89.6 | 88.9 | 87.2 |
| **Rajasthan** | 62.0 | 61.0 | 66.2 | 64.8 | 65.1 | 63.0 | 62.9 | 64.3 | 61.9 | 61.8 |
| **Uttar Pradesh** | DNA | DNA | DNA | 22.7 | 41.7 | 37.6 | 34.3 | 29.2 | 27.1 | 51.3 |
| **Uttarakhand** | 44.8 | 40.9 | 42.4 | 49.6 | 48.4 | 50.1 | 61.2 | 67.6 | 47.3 | 65.3 |
| **Andhra Pradesh** | 69.9 | 64.7 | 66.9 | 65.2 | 67.2 | 78.3 | 80.4 | 78.2 | 76.8 | 78.3 |
| **Karnataka** | 80.9 | 79.8 | 80.6 | 81.9 | 80.9 | 81.8 | 80.1 | 81.6 | 88.5 | 85.5 |
| **Kerala** | 95.5 | 95.7 | 96.5 | 96.7 | 96.8 | 95.8 | 96.1 | 96.4 | 98.4 | 95.8 |
| **Tamil Nadu** | 88.2 | 89.3 | 90.9 | 91.4 | 90.7 | 93.1 | 94.2 | 93.8 | 94.7 | 91.2 |
| **Goa** | 93.7 | 96.5 | 95.2 | 95.6 | 95.3 | 96.1 | 95.8 | 96.5 | 96.1 | 97.8 |
| **Gujarat** | 71.2 | 70.9 | 70.1 | 66.5 | 71.6 | 72.3 | DNA | DNA | 80.3 | 82.6 |
| **Maharashtra** | 81.8 | 82.7 | 78.8 | 80.2 | 80.9 | 81.2 | 81.1 | 83 | 80.3 | 79.9 |
| **Bihar** | 45.4 | 23.8 | DNA | DNA | 17.6 | 18.2 | 21.3 | 20.5 | 31.1 | 24.7 |
| **Jharkhand** | 51.4 | 47.1 | DNA | 42.7 | 45.9 | 52.2 | 60.9 | 52.4 | 48.6 | 39.7 |
| **Odisha** | 83.5 | 79.5 | 79.1 | 81.3 | 78.9 | 84.0 | 83.7 | 87.6 | 84.6 | 81.5 |
| **West Bengal** | 61.8 | 56.6 | 64.6 | 70.7 | 80.9 | 66.9 | 69.7 | 74.4 | 75.5 | 76.6 |
| **Chhattisgarh** | 53.6 | 51.9 | 51.3 | 62.5 | 65.9 | 75.6 | 72.8 | 80.8 | 80.2 | 75.5 |
| **Madhya Pradesh** | 56.8 | 53.1 | 57.3 | 59.3 | 61.9 | 60.0 | 49.4 | 51.7 | 56.1 | 60.7 |
| **Arunachal Pradesh** | 26.3 | 32.3 | 33.4 | 33.0 | 34.5 | 36.9 | 45.0 | 42.8 | 47.3 | 48.5 |
| **Assam** | 46.6 | 46.3 | 53.0 | 49.4 | 44.7 | 31.6 | 32.3 | 42.7 | 63.1 | 48.5 |
| **Manipur** | 42.2 | 39.9 | 40.8 | 41.1 | 40.4 | 42.4 | 36.2 | DNA | 44.9 | 34.6 |
| **Meghalaya** | 89.6 | 81.1 | 84.8 | 76.1 | 78.5 | 79.2 | 82.9 | DNA | 77.0 | 73.7 |
| **Mizoram** | 85.1 | 77.0 | 74.9 | 80.7 | 76.6 | 80.2 | 78.6 | 78.7 | 71.4 | 65.1 |
| **Nagaland** | 60.0 | 74.6 | 75.0 | 74.0 | 73.6 | 24.6 | 25.1 | 26.3 | 26.2 | 22.9 |
| **Sikkim** | 85.3 | 84.9 | 83.2 | 90.5 | 89.0 | 90.4 | 90.1 | 90.5 | 90.9 | 95.4 |
| **Tripura** | DNA | 39.6 | 44.8 | 35.8 | 61.5 | 36.6 | 41.4 | 39.4 | 70.8 | 71.3 |

DNA: Data Not Available

**Table A4: Socio-Demographic Index (SDI) Values for India and States, 2000-2018**

| **Location** | **2000** | **2001** | **2002** | **2003** | **2004** | **2005** | **2006** | **2007** | **2008** | **2009** | **2010** | **2011** | **2012** | **2013** | **2014** | **2015** | **2016** | **2017** | **2018** |
| --- | --- | --- | --- | --- | --- | --- | --- | --- | --- | --- | --- | --- | --- | --- | --- | --- | --- | --- | --- |
| **India** | 0.432 | 0.440 | 0.447 | 0.455 | 0.464 | 0.473 | 0.482 | 0.492 | 0.502 | 0.512 | 0.522 | 0.533 | 0.543 | 0.553 | 0.564 | 0.575 | 0.584 | 0.594 | 0.603 |
| **Andhra Pradesh** | 0.424 | 0.434 | 0.444 | 0.453 | 0.463 | 0.472 | 0.481 | 0.493 | 0.503 | 0.514 | 0.525 | 0.536 | 0.546 | 0.556 | 0.566 | 0.577 | 0.585 | 0.594 | 0.603 |
| **Arunachal Pradesh** | 0.413 | 0.422 | 0.430 | 0.438 | 0.447 | 0.457 | 0.466 | 0.477 | 0.487 | 0.500 | 0.513 | 0.526 | 0.539 | 0.552 | 0.565 | 0.578 | 0.589 | 0.600 | 0.611 |
| **Assam** | 0.421 | 0.429 | 0.437 | 0.446 | 0.454 | 0.462 | 0.471 | 0.479 | 0.487 | 0.496 | 0.504 | 0.514 | 0.523 | 0.532 | 0.542 | 0.552 | 0.562 | 0.571 | 0.580 |
| **Bihar** | 0.318 | 0.321 | 0.325 | 0.329 | 0.334 | 0.338 | 0.346 | 0.354 | 0.363 | 0.371 | 0.380 | 0.390 | 0.400 | 0.411 | 0.421 | 0.431 | 0.440 | 0.449 | 0.458 |
| **Chhattisgarh** | 0.376 | 0.386 | 0.395 | 0.405 | 0.414 | 0.425 | 0.436 | 0.449 | 0.461 | 0.473 | 0.485 | 0.497 | 0.509 | 0.521 | 0.534 | 0.547 | 0.558 | 0.568 | 0.579 |
| **Delhi** | 0.592 | 0.602 | 0.612 | 0.623 | 0.633 | 0.643 | 0.653 | 0.664 | 0.674 | 0.684 | 0.694 | 0.705 | 0.715 | 0.724 | 0.734 | 0.744 | 0.752 | 0.759 | 0.766 |
| **Goa** | 0.586 | 0.597 | 0.608 | 0.616 | 0.623 | 0.631 | 0.638 | 0.645 | 0.654 | 0.664 | 0.673 | 0.686 | 0.696 | 0.707 | 0.718 | 0.729 | 0.738 | 0.748 | 0.757 |
| **Gujarat** | 0.473 | 0.480 | 0.487 | 0.495 | 0.503 | 0.512 | 0.522 | 0.532 | 0.542 | 0.551 | 0.561 | 0.571 | 0.580 | 0.589 | 0.598 | 0.607 | 0.615 | 0.623 | 0.631 |
| **Haryana** | 0.484 | 0.494 | 0.503 | 0.513 | 0.524 | 0.534 | 0.545 | 0.557 | 0.569 | 0.580 | 0.591 | 0.602 | 0.613 | 0.624 | 0.635 | 0.646 | 0.655 | 0.664 | 0.674 |
| **Himachal Pradesh** | 0.496 | 0.508 | 0.520 | 0.531 | 0.541 | 0.553 | 0.564 | 0.575 | 0.587 | 0.598 | 0.610 | 0.622 | 0.633 | 0.644 | 0.654 | 0.662 | 0.669 | 0.677 | 0.685 |
| **Jammu and Kashmir** | 0.429 | 0.441 | 0.452 | 0.464 | 0.475 | 0.486 | 0.497 | 0.507 | 0.518 | 0.528 | 0.539 | 0.549 | 0.559 | 0.569 | 0.579 | 0.589 | 0.598 | 0.606 | 0.615 |
| **Jharkhand** | 0.365 | 0.373 | 0.381 | 0.389 | 0.397 | 0.406 | 0.415 | 0.424 | 0.433 | 0.442 | 0.453 | 0.464 | 0.475 | 0.487 | 0.499 | 0.512 | 0.523 | 0.534 | 0.545 |
| **Karnataka** | 0.457 | 0.465 | 0.474 | 0.481 | 0.490 | 0.499 | 0.509 | 0.521 | 0.532 | 0.543 | 0.554 | 0.565 | 0.576 | 0.586 | 0.596 | 0.606 | 0.614 | 0.622 | 0.631 |
| **Kerala** | 0.535 | 0.545 | 0.553 | 0.562 | 0.570 | 0.580 | 0.589 | 0.599 | 0.608 | 0.619 | 0.628 | 0.638 | 0.648 | 0.657 | 0.667 | 0.676 | 0.683 | 0.689 | 0.696 |
| **Madhya Pradesh** | 0.385 | 0.391 | 0.397 | 0.404 | 0.411 | 0.418 | 0.425 | 0.433 | 0.441 | 0.450 | 0.459 | 0.470 | 0.481 | 0.494 | 0.507 | 0.520 | 0.532 | 0.544 | 0.556 |
| **Maharashtra** | 0.512 | 0.519 | 0.526 | 0.534 | 0.543 | 0.553 | 0.564 | 0.576 | 0.588 | 0.599 | 0.610 | 0.620 | 0.630 | 0.639 | 0.648 | 0.658 | 0.666 | 0.674 | 0.682 |
| **Manipur** | 0.433 | 0.444 | 0.455 | 0.467 | 0.480 | 0.494 | 0.507 | 0.518 | 0.529 | 0.540 | 0.549 | 0.560 | 0.571 | 0.581 | 0.591 | 0.601 | 0.610 | 0.619 | 0.629 |
| **Meghalaya** | 0.402 | 0.417 | 0.431 | 0.443 | 0.455 | 0.467 | 0.477 | 0.488 | 0.498 | 0.508 | 0.519 | 0.530 | 0.541 | 0.552 | 0.563 | 0.575 | 0.585 | 0.595 | 0.605 |
| **Mizoram** | 0.482 | 0.492 | 0.502 | 0.511 | 0.520 | 0.528 | 0.537 | 0.545 | 0.553 | 0.562 | 0.572 | 0.581 | 0.591 | 0.601 | 0.611 | 0.621 | 0.630 | 0.640 | 0.649 |
| **Nagaland** | 0.456 | 0.473 | 0.490 | 0.505 | 0.519 | 0.533 | 0.545 | 0.559 | 0.572 | 0.585 | 0.597 | 0.610 | 0.622 | 0.634 | 0.645 | 0.654 | 0.661 | 0.669 | 0.676 |
| **Odisha** | 0.376 | 0.385 | 0.393 | 0.402 | 0.413 | 0.422 | 0.433 | 0.445 | 0.457 | 0.468 | 0.480 | 0.491 | 0.503 | 0.514 | 0.526 | 0.537 | 0.547 | 0.557 | 0.567 |
| **Punjab** | 0.503 | 0.511 | 0.519 | 0.527 | 0.535 | 0.545 | 0.555 | 0.566 | 0.576 | 0.587 | 0.598 | 0.608 | 0.618 | 0.627 | 0.636 | 0.643 | 0.650 | 0.657 | 0.663 |
| **Rajasthan** | 0.382 | 0.390 | 0.397 | 0.405 | 0.414 | 0.422 | 0.430 | 0.440 | 0.449 | 0.457 | 0.468 | 0.479 | 0.490 | 0.500 | 0.511 | 0.521 | 0.530 | 0.538 | 0.547 |
| **Sikkim** | 0.430 | 0.441 | 0.453 | 0.466 | 0.478 | 0.490 | 0.501 | 0.511 | 0.520 | 0.536 | 0.551 | 0.565 | 0.579 | 0.593 | 0.607 | 0.620 | 0.632 | 0.643 | 0.655 |
| **Tamil Nadu** | 0.487 | 0.496 | 0.505 | 0.513 | 0.521 | 0.531 | 0.541 | 0.552 | 0.563 | 0.575 | 0.587 | 0.599 | 0.610 | 0.621 | 0.632 | 0.640 | 0.648 | 0.655 | 0.662 |
| **Telangana** | 0.417 | 0.428 | 0.438 | 0.449 | 0.459 | 0.470 | 0.482 | 0.495 | 0.509 | 0.521 | 0.535 | 0.549 | 0.562 | 0.574 | 0.587 | 0.599 | 0.609 | 0.620 | 0.630 |
| **Tripura** | 0.438 | 0.451 | 0.462 | 0.470 | 0.478 | 0.485 | 0.491 | 0.497 | 0.504 | 0.511 | 0.518 | 0.527 | 0.536 | 0.545 | 0.554 | 0.564 | 0.573 | 0.581 | 0.590 |
| **Uttar Pradesh** | 0.362 | 0.368 | 0.374 | 0.382 | 0.390 | 0.398 | 0.407 | 0.415 | 0.423 | 0.432 | 0.441 | 0.451 | 0.462 | 0.473 | 0.485 | 0.498 | 0.508 | 0.519 | 0.530 |
| **Uttarakhand** | 0.412 | 0.424 | 0.436 | 0.449 | 0.463 | 0.477 | 0.491 | 0.505 | 0.519 | 0.535 | 0.551 | 0.568 | 0.583 | 0.598 | 0.613 | 0.628 | 0.639 | 0.651 | 0.663 |
| **West Bengal** | 0.438 | 0.446 | 0.454 | 0.462 | 0.470 | 0.478 | 0.486 | 0.496 | 0.506 | 0.516 | 0.526 | 0.536 | 0.546 | 0.555 | 0.565 | 0.573 | 0.580 | 0.588 | 0.595 |
| **Standard deviation** | 0.064 | 0.065 | 0.066 | 0.066 | 0.066 | 0.067 | 0.067 | 0.068 | 0.068 | 0.069 | 0.069 | 0.069 | 0.069 | 0.069 | 0.068 | 0.068 | 0.067 | 0.066 | 0.066 |
| **Standard deviation /**  **Average SDI** | 14.4% | 14.3% | 14.2% | 14.0% | 13.8% | 13.7% | 13.5% | 13.3% | 13.1% | 12.9% | 12.7% | 12.5% | 12.2% | 11.9% | 11.6% | 11.3% | 11.0% | 10.8% | 10.5% |

Global Burden of Disease Collaborative Network: **Global Burden of Disease Study 2016 (GBD 2016) Socio-Demographic Index (SDI) 1970–2016.** In *Global Burden of Disease* (Insitute of Health Metrics and Evaluation (IHME) ed. Seattle; 2017.
